# Anesthesia and Cancer, Friend or Foe? A Narrative Review

**DOI:** 10.3389/fonc.2021.803266

**Published:** 2021-12-23

**Authors:** Julio Montejano, Vesna Jevtovic-Todorovic

**Affiliations:** School of Medicine, University of Colorado, Aurora, CO, United States

**Keywords:** cancer recurrence, metastatic conversion, general anesthesia, regional anesthesia, total intravenous anesthesia, dexmedetomidine, lidocaine infusion

## Abstract

Cancer remains the leading cause of death worldwide with close to 10 million deaths reported annually. Due to growth of the advanced age cohort in our population, it is predicted that the number of new cancer cases diagnosed between now until 2035 is to reach potentially 24 million individuals, a staggering increase in a relatively short time period. For many solid tumors, surgical resection along with chemotherapy is the best available approach to a potential cure which leads to almost 80% of cancer patients undergoing at least one surgical procedure during the course of their disease. During surgical intervention, the exposure to general anesthesia can be lengthy, complex and often involves various modalities resulting in an important question as to the role, if any, anesthesia may play in primary recurrence or metastatic conversion. Many components of the stress and inflammatory responses exhibited in the perioperative period can contribute to cancer growth and invasion. The agents used to induce and maintain general anesthesia have variable interactions with the immune and neuroendocrine systems and can influence the stress response during surgery. Thus, debating the best type of anesthesia that would help to attenuate sympathetic and/or pro-inflammatory responses while modulating cytokine release and transcription factors/oncogenes remains at the forefront. This may affect inducible cancer cell survival and migratory abilities not only intra-operatively, but also during the immediate post-operative phase of recovery. The ultimate question becomes how and whether the choice of anesthesia may influence the outcomes of cancer surgery with two major approaches being considered, i.e., regional and general anesthesia as well as the various hypnotics, analgesics and sympatholytics commonly used. In this review, we will address the latest information as to the role that anesthesia may play during cancer surgery with specific focus on primary recurrence and metastasis.

## Introduction

Although more is known about cancer biology and treatment today than ever before, cancer remains the leading cause of death worldwide and it is predicted that this death toll will only continue to increase owed to our ever-aging population (1–3). The perioperative period presents a unique conundrum for the perioperative care team; patients present for surgery to be cured of their disease and yet find themselves at risk of recurrence and metastatic conversion, two major sources of morbidity for patients having tumor resection with curative intent (3). The perioperative period is well known for activating the body’s natural stress response starting with upregulated neuroendocrine signaling, increased release of pro-inflammatory mediators and heightened immunomodulation (4). Additionally, surgical resection of solid tumors leads to increased sympathetic output and invites a pro-inflammatory response to tissue damage which is necessary for tissue repair and healing. This biological response to surgical stress can be hijacked and used for the benefit of any remaining cancer cells to ensure their survival and possibly allow them to migrate. Metastatic disease is the most common cause of death for cancer patients and it can be a source of great financial burden and emotional distress for patients and their families (5).

The biology of cancer cell survival and migration in the perioperative period is frequently studied and is extremely complex (4); hence, much time and effort have been spent to examine two important considerations, the effects of anesthetic techniques and drug choices on the risk of primary recurrence and metastatic conversion for these patients. To date there have been numerous *in vitro*, *in vivo* and retrospective studies as well as several prospective randomized controlled trials performed in hopes of addressing these considerations (2, 4, 6–14). In this review, we will report the latest data investigating the role of anesthesia in cancer recurrence and metastatic conversion. It is important to note that to date, there are no official recommendations for best practice in this area. Many studies have suggested some anesthetic agents have the potential to be harmful and increase the risk of recurrence or disease progression while others have been shown to decrease these risks.

A literature search was performed using public databases with the following key words: cancer recurrence, metastatic conversion, general anesthesia, regional anesthesia, total intravenous anesthesia, dexmedetomidine and systemic local anesthetics. Appropriateness for inclusion in the narrative was determined by the authors to include a wide and unbiased range of recent and pertinent studies. Thus in order to examine how anesthesia may affect patient outcomes we will discuss basic tumor biology and some of the potential targets available for modulation. Then we will report and comment on recent studies comparing outcomes for patients undergoing solid tumor resections under general anesthesia *vs* regional anesthesia followed by outcome data comparing two major types of general anesthesia—volatile anesthesia (e.g., isoflurane, sevoflurane, desflurane) *vs* propofol based TIVA (Total Intravenous Anesthesia). Lastly, some recent data on the oncogenic effects of various commonly used anesthetic agents will be discussed.

## Current State

There have been tremendous advances in the field of cancer biology over the past decade and though our understanding has deepened, there is much that remains a mystery (1). Factors affecting cancer recurrence and metastatic conversion at the time of primary resection are two facets of cancer biology that remain incompletely understood.

There are three basic mechanisms by which recurrence and metastatic conversion occur (15, 16). The first is local recurrence where surviving cancer cells may proliferate at the primary site of resection *via* mechanisms involving pro-inflammatory cytokines, pro-oncogenes and angiogenic factors. Second, cancer cells may transform and acquire the ability to travel to distant sites through either vascular or lymphatic spread due activation and mutation of oncogenes. And third, body cavity seeding during primary tumor resection. The use of intraperitoneal chemotherapy during cytoreductive surgery or primary resection of cancer is one tool aimed at destroying microscopic disease (17–19).

As previously noted, surgical resection of tumors induces an expected state of systemic inflammation and local hypoxia as a result of tissue damage and immunomodulation that may facilitate the conversion of solid tumors into metastatic disease, otherwise known as the epithelial to mesenchymal conversion (20–22). At the same time this pro-inflammatory state exerts a myriad of effects on the body’s own cell mediated immune response. There is an intricate interplay between the release of cortisol and catecholamines and the function of immune cells including but not limited to natural killer (NK) cells and CD8+ T cells, both of which are stunted in their antitumor activity. Additionally pro-oncogenic cell lines, regulatory T cells and type 2 helper T cells are activated and encouraged to proliferate in such a state (4).

It is therefore only logical that anesthesiologists would look to take advantage of the sympatholytic, anti-inflammatory and immunomodulatory effects of anesthetic drugs in an attempt to modify this process and improve patient outcomes. In essence, the ideal anesthetic for cancer patients would:

- Attenuate sympathetic response while maintaining adequate tissue perfusion to avoid tissue hypoxia
- Attenuate pro-inflammatory milieu while maintaining an adequate healing response
- Modulate cytokine release and cellular function to lean toward promoting NK and CD8+ cell activity
- Modulate transcription factors and oncogenes to prevent inducible cell survival and migration

Unfortunately, despite promising *in vitro* and *in vivo* studies it appears that this process is far more complex than originally thought, likely owed to both the heterogeneous biology of different malignancies and patient populations. Recent prospective randomized clinical trials (RCTs) have shown little promise at elucidating the perfect anti-oncogenic anesthetic, however there are dozens of active multicenter RCTs aimed at shedding light on this topic (1, 23).

## Use of General Anesthesia *vs* Regional Anesthesia

Volatile anesthetics and other hypnotics used to induce and maintain general anesthesia have several anti-inflammatory and immunomodulatory effects (2, 24–30). Regional anesthetic techniques, ranging from peripheral nerve blocks to neuraxial analgesia, are already employed in many primary tumor resections in order to reduce post-surgical pain and decrease opioid consumption (31–35). From a physiologic point of view, it is logical that one would expect an improvement in recurrence or conversion outcomes, owed to the powerful sympatholytic effects of regional anesthesia in addition to avoidance of the potentially detrimental immunosuppressive effects of volatile anesthetics and opioids. In 2019, one of the largest RCTs to date, evaluated the use of paravertebral nerve blocks (PVB) combined with propofol TIVA in women undergoing primary mastectomy for breast cancer and compared it to volatile anesthesia and conventional opioid analgesia (25). Recurrence occurred in 102 (9.8%) *vs* 111 (10.4%) women in the regional anesthesia *vs* volatile general anesthesia groups, which was found to be statistically significant and passed the study’s futility threshold. The study was aborted at that time and no further data was collected. In this study, it was concluded that the use of regional-propofol anesthesia does not impact breast cancer recurrence (25). Although, this study was appropriately powered and the results seem compelling, we must not forget about the extreme heterogeneity of oncologic disease and should apply caution when generalizing studies such as this to other patient populations. More studies are needed in order to definitively recommend regional *vs* general anesthesia for any given malignancy or patient population. Although with recent advances in surgical technique more and more surgeries can be performed under regional anesthesia (36) it should be noted that nearly all oncological surgeries require general anesthesia in order to be feasible and safe.

## TIVA *vs* Volatile Gas Anesthesia

An interesting question remains whether the known effects of volatile anesthetics on immune function are detrimental for cancer recurrence and metastatic conversion. *In vitro and in vivo* studies have shown that when breast, ovarian and renal cell carcinoma cells are exposed to volatile gases there is increased cytokine release (IL-1/6/8 and TNF), NK and T-cell modulation as well as an increase in growth, angiogenic and migration factors (3, 7, 37–39). However, for other cancer types such as non-small cell lung cancer (NSCLC) exposure to volatile anesthetics has been shown to be suppressive of growth and migration (40). The Cancer and Anesthesia Study (CAN NCT01975064), one of the largest RCTs to study recurrence and survival in breast cancer patients following exposure to general anesthesia, recently published its analysis of first year survival data for 1705 patients with breast cancer (41). These patients were randomized to either a volatile anesthetic *vs* TIVA with propofol and no difference in survival was observed between the two groups at one year; patients will continue to be followed until 2022. The CAN trial contains two other arms which include patients undergoing primary resection of colorectal cancer which are still in progress. This study points to some important complexities which include the heterogeneity of tumor biology including different cancer types, length of surgery and patient factors such as race and other environmental factors. It was noted in this study that patients of Chinese descent had improved survival rates at one year than other groups (41). To date there has been one RCT that showed propofol decreased local recurrence of breast intraductal carcinoma for patients undergoing primary resection with the goal of breast conservation (42). This study included 2036 women of Asian descent randomized to receive either propofol TIVA and PVB *vs* volatile anesthesia and PVB. Women who received propofol showed a significant reduction in local recurrence risk; however, there was no difference in risk of metastatic conversion. In short, more data is needed to definitively say whether exposure to one type of anesthetic is beneficial or harmful for the survival of cancer patients.

## Opioids

Due to the world-wide opioid epidemic, the use of opioids in anesthesia has long been under question as there are more and more pharmacologic agents that can be used to manage intraoperative and post-operative pain as well as achieve sympatholysis during general anesthesia. Opioids are powerful immunomodulators which are known to affect innate cell immunity by downregulating NK cell activity and decreasing cytokine production (31, 43). This effect is thought to be due to mu-opioid receptor activity as evidenced in one study by improved survival in colorectal and breast cancer patients receiving mu-opioid receptor antagonists, such as naloxone (44, 45). Other cell and animal studies have shown that opioids have a direct effect on tumor growth *via* activation of transcription factors (46). Additionally, opioids have been shown to be pro-angiogenic through activation of VEGF-receptors (30, 45, 47). For decades it was thought that opioids were largely ubiquitous in their immunomodulatory effects and morphine was used as the prototypical opioid profile; however, with recent data it is becoming clear that different opioids exert different effects on the immune system. For example, morphine and fentanyl have been shown to have similar effects on NK-cell activity and lymphocyte proliferation; however, oxycodone has been shown to have minimal immunosuppressive properties (48). Despite this data, it would be naïve to think that it might be possible to completely eliminate the use of opioids in the treatment of pain in cancer patients as they are the most commonly employed analgesic drugs in the post-operative period (33). Frustratingly, opioid sparing techniques do not seem to affect short term survival as noted in one study that randomized patients to receive remifentanil infusions (47). Thus, the question becomes whether there is a balance of pharmacologic effects between anesthetic and analgesic agents that could be found to improve patient disease free survival.

## Alpha-2 Agonists

Clonidine and dexmedetomidine are powerful α2-adrenoceptor agonists used in general anesthesia and ICU care for their analgesic effects, opioid sparing properties as well as powerful sedative and anxiolytic effects. Some studies have found dexmedetomidine to be neuroprotective and an improvement in postoperative cognitive dysfunction through reduction of serum TNF-α, IL-6, PI3K and AKT, which would also suggest that dexmedetomidine is anti-inflammatory (49–51). Because of its analgesic properties and excellent performance as a sympatholytic, dexmedetomidine is an alluring choice for use in general anesthesia for cancer patients. Even when compared to clonidine, dexmedetomidine is significantly more efficacious with fewer side effects. There is however, much evidence to suggest the contrary.

There have been numerous *in vivo* and *in vitro* studies showing that dexmedetomidine may in fact increase the risk for recurrence by modulating cell survival through activation of HIF-1α as well as increased secretion of metalloproteinases (MMP) which have been implicated in cell migration and metastatic conversion (6, 9–11, 14, 38, 50, 52–56). The transcription factor HIF-1 α has been shown to confer a survival advantage to cells when exposed to hypoxic conditions, such as when vascular supply is removed during resection (9, 52, 54, 56). Bruzzone et al. first found that α2-adrenoceptors have a positive effect on the proliferation of a mouse mammary tumor cell line *in vitro (* 57). In addition to already discussed effects of HIF-1α, dexmedetomidine induces the proliferation of myeloid-derived suppressor cells associated with significant proangiogenic potential, promoting tumor metastasis through increasing production of VEGF (9). Furthermore, dexmedetomidine upregulates the expression of survivin, MMP−2, MMP−9, all implicated in metastatic conversion of lung adenocarcinoma (56). A recent retrospective study for patients with NSCLC showed that the use of dexmedetomidine had no benefit on recurrence free survival and a significantly lower overall-survival for patients who underwent primary surgical resection (56). These effects have been noted in other cancer types such as esophageal, colorectal and hepatocellular carcinoma (6, 7).

As with previous hypotheses involving the effects of anesthesia on cancer recurrence and metastatic conversion these data are not practice altering. Quality evidence in support of or against use of dexmedetomidine in clinical practice for cancer patients is lacking. More prospective RCTs are needed to determine whether effects seen in cell and animal studies will pan out. However, with the number of studies suggesting potential harm from dexmedetomidine it is probably prudent to avoid using it if “safer” alternatives are available. There are several RCTs aimed at studying the effects of dexmedetomidine on cancer recurrence. One trial examining the impact of dexmedetomidine on breast cancer recurrence is due to be completed in 2024 (NCT03109990) (23).

## Local Anesthetics

Amide local anesthetics, specifically lidocaine, have long been a useful tool in the management of pain during general anesthesia, employed both as systemic intravenous infusions and during neuraxial and peripheral nerve blocks. Lidocaine is a short acting minimally toxic sodium channel blocker that acts to decrease nerve conduction and results in reduction of pain scores in patients receiving intravenous infusions intraoperatively and postoperatively (35, 58–60). In addition to its analgesic properties, lidocaine exhibits anti-oncogenic and anti-inflammatory effects through various pathways (61–64). Dozens of laboratory studies have been performed to flesh out the biological pathways responsible for lidocaine’s observed effects (12, 13, 32, 65–68). Unfortunately, clinical data including retrospective analyses are sparce. To date there has been one study reporting on the observed clinical effects of lidocaine on recurrence of pancreatic cancer, which showed that patients treated with intravenous infusions of lidocaine had better survival rates at 1 and 3 years with no difference in disease-free survival (65). These anti-inflammatory effects have been observed even through alternative methods of local anesthetic administration including intraperitoneal lavage. In one prospective randomized controlled study of patients undergoing ovarian tumor resection, it was observed that patients who received intraperitoneal washings of ropivacaine had a shortened time to chemotherapy administration vs patients in the placebo arm (69). Though the mechanism by which this was achieved is unclear, the authors proposed it could be due to an attenuated inflammatory response, local anesthetic cytotoxicity for microscopic disease in the peritoneum and improved wound healing. While not a direct effect on cancer recurrence, the effect noted in this study could suggest an alternative use for local anesthetics that could hasten a patient’s treatment course—several studies have concluded that early chemotherapy administration is associated with improved outcomes although the timeline is still under debate (70).

An upcoming RCT, Volatile Anesthesia and Perioperative Outcomes Related to Cancer trial (VAPOR-C, NCT04316013) set to complete in 2025, will examine the effects of lidocaine in patients with lung or colorectal adenocarcinoma (71). It is important to remember that using intravenous lidocaine as an analgesic is off-label. To date there have been no studies to show that lidocaine infusions are harmful to cancer patients so long as they are employed judiciously and there are no contraindications or conditions that would increase toxicity, such as severe liver disease or low protein states (32, 60, 61, 71). Centers using intravenous lidocaine infusions should have safety protocols and dosing guidelines to avoid harm in patients receiving this treatment (60). Time and care should be applied to training personnel in the recognition of lidocaine toxicity and treatment both intraoperatively and in the post-operative period (61). At our institution, it is common to use intravenous lidocaine infusions for patients that have undergone colorectal surgery, not always to treat oncologic disease, as part of an enhanced recovery after surgery (ERAS) protocol to aid in gut motility and decrease opioid consumption.

## Conclusion

**Figure 1:**
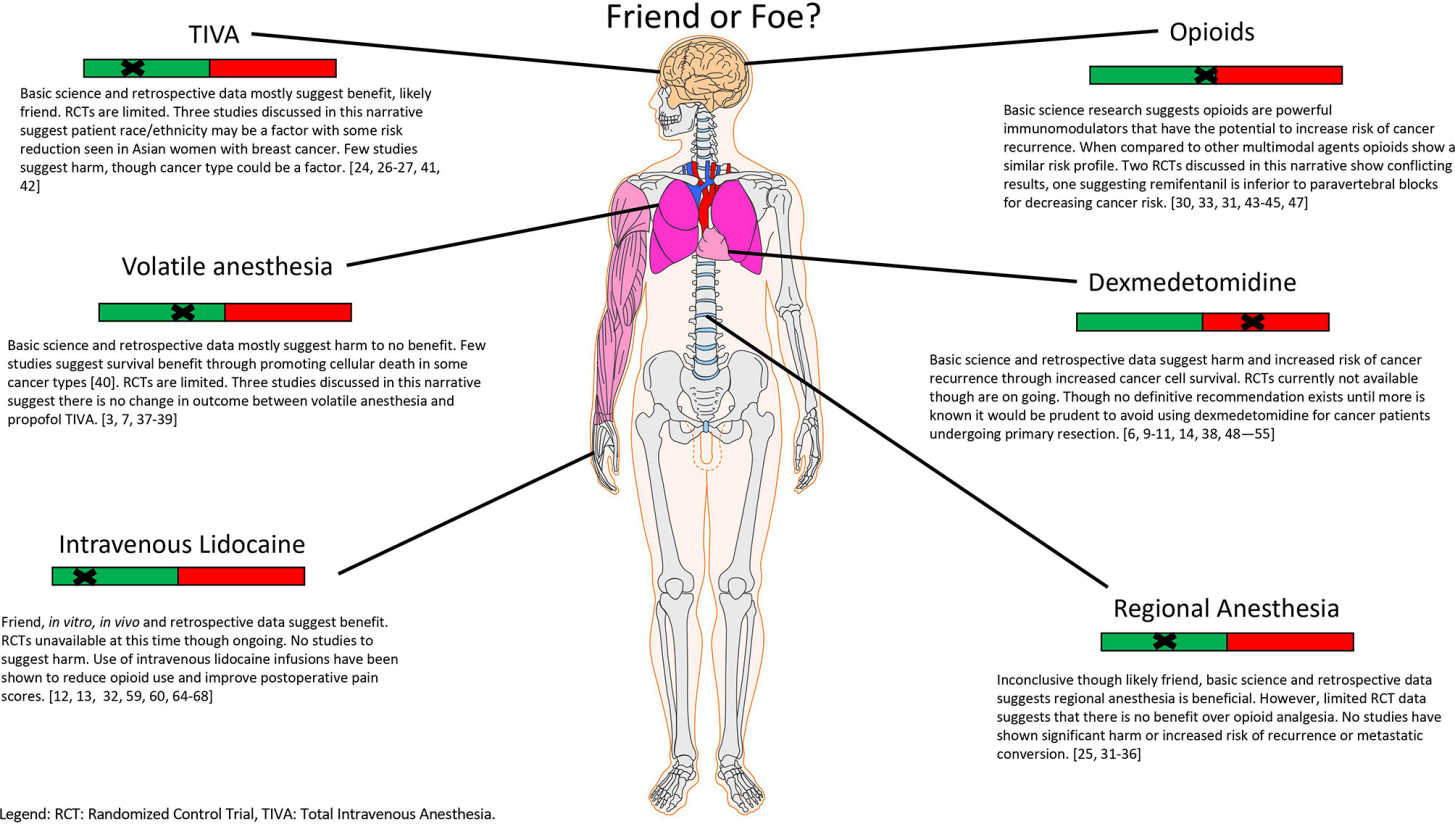
A summary of findings depicted in this narrative review.

Optimization of the care of cancer patients is in constant flux and evolution. The perioperative period has been identified as a unique intersection of intent to treat with potential harm coming to the patient due to that treatment. Anesthesiologists and surgeons are in the unique position to affect a patient’s postoperative course and survival outcome. Specifically, the agents chosen to induce and maintain general anesthesia while surgical intervention is performed have the potential to bring benefit or harm to these patients (**Figure 1**). In this review, we have briefly discussed cancer cell biology and how recurrence and metastatic conversion may occur as a result of the interplay between circulating tumor cells, cytokines, the HPA axis, the immune system, growth and migration factors and catecholamines as well as the effects of several commonly used hypnotics and analgesics. Despite the numerous studies performed to date, the data currently available is insufficient to form a definitive recommendation for anesthetic choice. Although, from a mechanism point of view, it is tempting to hope that the perfect anesthetic exists for mitigating the risk of cancer recurrence given the vast complexity of oncologic disease and patient genetic heterogeneity it is likely that we may never have an answer. It will likely require genetic phenotyping of patients and their disease to personalize the delivery of anesthesia, while the technology is available it is far from being applied clinically (72–74). Nevertheless, given the immense impact that oncologic disease has worldwide and that it is only projected to continue to worsen this remains an area of high potential for improving the lives of many.

## Author Contributions

JM was the first author contributing literature review, writing manuscript as well as design of figure. VJ-T is the senior author contributing expertise in the field of anesthesiology, editing and advising as well as writing of abstract. All authors contributed to the article and approved the submitted version.

## Conflict of Interest

The authors declare that the research was conducted in the absence of any commercial or financial relationships that could be construed as a potential conflict of interest.

## Publisher’s Note

All claims expressed in this article are solely those of the authors and do not necessarily represent those of their affiliated organizations, or those of the publisher, the editors and the reviewers. Any product that may be evaluated in this article, or claim that may be made by its manufacturer, is not guaranteed or endorsed by the publisher.
